# *Euphonic*: inelastic neutron scattering simulations from force constants and visualization tools for phonon properties

**DOI:** 10.1107/S1600576722009256

**Published:** 2022-11-29

**Authors:** Rebecca Fair, Adam Jackson, David Voneshen, Dominik Jochym, Duc Le, Keith Refson, Toby Perring

**Affiliations:** a ISIS Neutron and Muon Source, STFC Rutherford Appleton Laboratory, Didcot OX11 0QX, UK; b Scientific Computing Department, STFC Rutherford Appleton Laboratory, Didcot OX11 0QX, UK; c Department of Physics, Royal Holloway University of London, Egham TW20 0EX, UK; Australian Nuclear Science and Technology Organisation, Lucas Heights, Australia

**Keywords:** neutron scattering, phonons, force constants, *Euphonic*

## Abstract

Interpretation of vibrational inelastic neutron scattering spectra of complex systems is frequently reliant on accompanying simulations from theoretical models. *Ab initio* codes can routinely generate force constants, but additional steps are required for direct comparison with experimental spectra. On modern spectrometers this is a computationally expensive task due to the large data volumes collected. In addition, workflows are frequently cumbersome as the simulation software and experimental data analysis software often do not easily interface to each other. Here a new package, *Euphonic*, is presented. *Euphonic* is a robust, easy to use and computationally efficient tool designed to be integrated into experimental software and able to interface directly with the force constant matrix output of *ab initio* codes.

## Abstract 2

An overview of the *Euphonic* Python package for efficient simulation of inelastic neutron scattering by phonons from force constants is presented.

## 1. Introduction

The study of atomic vibrations is of both fundamental and applied interest as they have a significant role in many macroscopic material properties. They can drive phase transitions (Budai *et al.*, 2014 ▸), transport heat (Zheng *et al.*, 2021 ▸), govern elastic properties (Böer & Pohl, 2018 ▸) and even limit the coherence of qubits (Garlatti *et al.*, 2020 ▸). For this reason significant effort has been devoted to understanding atomic vibrations both theoretically and experimentally. Inelastic neutron scattering (INS) is a particularly powerful experimental technique, as the momentum and frequency dependence of the atomic vibrations can be straightforwardly and quantitatively related to the experimental intensities, providing a stringent test of theoretical models.

The earliest experiments were performed using a triple-axis spectrometer (Brockhouse, 1955 ▸) and this remains a widely applied method. In its conventional form this instrument defines the incident (final) neutron energy with a crystal monochromator (analyser) and a single detector measuring at a single point in **Q**–ω (momentum–energy) space. Dispersion curves are mapped out by making a series of measurements as a function of energy transfer at fixed momentum, or as a function of momentum at fixed energy transfer, which collectively raster over a **Q**–ω region of interest. To improve efficiency, additional analyser–detector groups after the sample can be used (Kempa *et al.*, 2006 ▸), increasing data rates but also increasing the volume and complexity of the data to be modelled.

Alternatively, a time-structured beam may be used in conjunction with beamline components that define the incident neutron energy or final neutron energies, so that each detector in an array can map out many different energy transfers ω. These time-of-flight (TOF) instruments have become increasingly popular, especially with the advent of high-power pulsed spallation sources. To maximize their efficiency, these instruments have detector arrays which cover large solid angles broken up into pixels. For example, LET at ISIS (Bewley *et al.*, 2011 ▸) has a three-steradian detector array that is split into 98 304 pixels, in each of which the energy transfer range is resolved into ∼300 bins. For single-crystal experiments the sample must be rotated, commonly in 0.5–1° steps over a 180° range, leading to the generation of a multidimensional data set which consists of 10^9^–10^10^ individual **Q**–ω points. Data analysis frameworks to handle and interact with these objects exist and are widely used (Ewings *et al.*, 2016 ▸; Reznik & Ahmadova, 2020 ▸; Arnold *et al.*, 2014 ▸).

The calculation of vibrational properties from first principles or parameterized atomistic lattice dynamics is well established within the computational chemistry and physics communities. As the equations needed are straightforward, many codes have been written to compute INS spectra for comparison between theory and experiment. These include *SimPhonies* (Bao *et al.*, 2016 ▸), *OpenPhonon* (Mirone & d’Astuto, 2006 ▸), *Scatter* (Roach *et al.*, 2007 ▸) for *GULP* (Gale, 2005 ▸), *ab2tds* (Mirone & Wehinger, 2013 ▸), the *CLIMAX* series (Kearley & Tomkinson, 1990 ▸; Ramirez-Cuesta, 2004 ▸; Cheng *et al.*, 2019 ▸) and *PHONON* (Parlinski, 2014 ▸), in addition to private and unreleased codes. Recent versions of *Phonopy* (Togo & Tanaka, 2015 ▸) also include functions for calculation of the dynamical structure factor. However, no available software can meet all of the requirements of a tool suitable for experimenters at TOF INS facilities, as each program lacks one or more functionalities such as flexible and customizable modelling of instrumental resolution; scaling of computational performance to very large high-resolution data sets; ease of use; and availability of maintained source code.

The force constants approach of the above-mentioned codes is restricted to systems and excitations well described by the harmonic approximation of lattice dynamics. Anharmonic phenomena including frequency shifts of asymmetric bond vibrations and overtones, broadening, dynamically stabilized and high-temperature structural phases, and nuclear quantum dynamics are poorly described or not included at all. In some cases it is feasible to still compute stable harmonic phonons using volume- or temperature-dependent effective potentials (Hellman *et al.*, 2013 ▸). Computational approaches to strong anharmonicity are not as well established or widespread, but one which merits mention in the context of INS spectra is based on the analysis of dynamical correlation functions computed using molecular dynamics simulations. The software packages *nMoldyn* (Róg *et al.*, 2003 ▸), *MDANSE* (Goret *et al.*, 2017 ▸), *Dynasor* (Fransson *et al.*, 2021 ▸) and *LiquidLib* (Walter *et al.*, 2018 ▸) all perform calculation of dynamical structure factors from molecular dynamics trajectory data.

Here we present *Euphonic* (Fair *et al.*, 2022*a* ▸), a software package to calculate INS intensities in the harmonic approximation using force constants obtained from *ab initio* calculations. It is written in Python, for ease of integration with other software and experimental workflows. This allows it to be used, for example, to simulate any part of a TOF **Q**–ω data set with instrumental resolution convolution via the data analysis package *Horace* (Ewings *et al.*, 2016 ▸). Given the potential size of the **Q**–ω TOF data sets described above, *Euphonic* has a focus on computational performance and uses an extension written in C and OpenMP to handle the most demanding steps. *Euphonic* is not strongly coupled to any particular atomistic code; currently, force constants can be read from *CASTEP* (Clark *et al.*, 2005 ▸), which internally implements several schemes for phonon calculations, and *Phonopy* (Togo & Tanaka, 2015 ▸), which manages finite-displacement calculations with any of 11 force calculators. In addition to simulating large **Q**–ω TOF data sets, it is intended as a general-purpose tool for analysis of phonon simulations. Command-line programs are included for quick calculation and plotting of phonon band structure, density of states (DOS) and the neutron dynamical structure factor, while the Python API allows *Euphonic* to be used as a more flexible library for optimized phonon frequency and eigenvector calculations and related quantities.

In this paper we describe the basic theory behind *Euphonic*; the software structure, its main features and how it interacts with other software; we show how its performance and results compare with similar existing software tools; and, finally, we compare *Euphonic* with experimental data sets for both single crystals and powders.

### 1.1. Availability

*Euphonic* has been developed following software development best practice: continuous integration processes test the functionality and validate numerical results as features are added, and the Python API has been explicitly designed to make *Euphonic* easy to use with other projects. *Euphonic* is open source under the GNU General Public License v3; the source code is freely available on Github, and packages are distributed by *PyPI* and *Conda-forge* for the *pip* and *conda* package managers. Links to the source code and online documentation, including instructions on installation and how to use *Euphonic*, are available at https://doi.org/10.5286/SOFTWARE/EUPHONIC (Fair *et al.*, 2022*a* ▸).

### 1.2. Validation data sets

Throughout this paper a variety of simulated and experimental data sets will be used to compare *Euphonic* outputs with both software tools and experimental data. The chosen materials are Nb, Al, Si, quartz and La_2_Zr_2_O_7_ and will be described and used in each section as appropriate. These data sets have been chosen to give a variety of unit-cell sizes and crystal symmetries, and include both polar (quartz) and non-polar materials. Simulated force constants have been produced with either *CASTEP* or *VASP* (Kresse & Furthmüller, 1996 ▸) plus *Phonopy* in order to thoroughly test the features of *Euphonic*. Details of the force constant calculations for each material and where to obtain the results of the simulations and the neutron experimental data can be found in the supporting information.

## 2. Theory

### 2.1. Harmonic lattice dynamics

Vibrational excitations – phonons – in crystalline materials are described within the theory of lattice dynamics (Dove, 1993 ▸). Displacement of a single atom in an infinite periodic crystal gives rise to forces on every other atom in the crystal, which in the harmonic approximation are linearly related to the displacement by the force constant matrix 

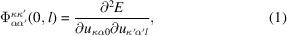

where Φ is the force constant matrix, the unit cell containing the displaced atom is labelled 0, *l* runs over unit cells in the crystal, κ runs over atoms in the unit cell, α runs over the Cartesian directions, 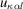 is the displacement of atom κ in cell *l* in direction α from its equilibrium position, and *E* is the total lattice energy. These force constants decay rapidly with distance according to a power law |*R*|^−*n*^, where *n* = 5–7 in non-polar crystals. This allows for a truncation of Φ(0, *l*) at some radius *R*_c_ beyond which the residual force constants may be neglected. *R*_c_ ≃ 8–10 Å is sufficient for almost all practical cases.

Substituting a plane-wave solution into the equation of motion yields the eigenvalue equation 

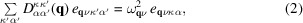

where 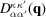 is the dynamical matrix at wavevector **q**, the eigenvalues 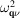 are the square of the phonon frequencies of mode ν at **q**, and the eigenvectors 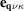 are the phonon polarization vectors at **q** of mode ν for atom κ. The dynamical matrix can be calculated as the mass-weighted Fourier transform of the force constant matrix: 

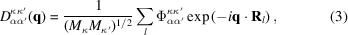

where *M*_κ_ is the mass of atom κ, **R**_*l*_ is the vector from the origin to the *l*th unit cell and *l* includes unit cells with |**R**_*l*_| < *R*_c_.

#### 2.1.1. Deconvolution of periodic representation of Φ

Though the force constant matrix Φ(0, *l*) is a finite matrix, it does not map directly onto a modelling framework with supercell periodic boundary conditions as used in almost all *ab initio* lattice dynamics implementations. Instead these yield a convolution of Φ(0, *l*) with the lattice of a suitable supercell. Ideally this would be chosen so the magnitude of Φ(0, *l*) falls to a negligible value in half the supercell linear dimension to avoid overlap error. The force constants may be computed either directly, using the supercell as the *ab initio* simulation cell (the ‘direct method’) (Ackland *et al.*, 1997 ▸), or implicitly, using Fourier interpolation of either density-functional perturbation theory (Baroni *et al.*, 2001 ▸) or finite displacements (Ackland *et al.*, 1997 ▸). Therefore *Euphonic* must deconvolve a periodic data set to recover the aperiodic force constant matrix Φ(0, *l*). For a very large supercell the supercell-periodic images of Φ(0, *l*) truncated at some radius *R*_c_ do not overlap at all and the extraction is a straightforward mapping of the data values. However in practical *ab initio* calculations the supercell sizes are limited by computational resources, and there is always a small residual overlap between periodic images of Φ(0, *l*). *Euphonic* adopts the ‘cumulant image’ approach (Parlinski *et al.*, 1997 ▸), combining deconvolution with Fourier transform to compute the dynamical matrix in a formulation which respects point-group symmetry.

#### 2.1.2. Acoustic sum rule

The invariance of the total energy on displacement of the entire crystal in space imposes a condition known as the acoustic sum rule, which guarantees the presence of the usual three acoustic modes with zero frequency at **q** = 0 and linear dispersion nearby. The sum rule applies to the force constant matrix or the Γ-point dynamical matrix: 

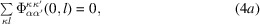




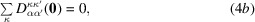

where κ runs over *N* atoms in the unit cell and *l* over the *N*_cell_ unit cells in the supercell. The zeros depend on an exact cancellation of an entire row of the dynamical or force constant matrices, whose elements are large and of opposite sign. In the presence of numerical convergence errors and symmetry breaking by typical *ab initio* computational grids, the sum rule is not exactly satisfied, leading to non-zero acoustic modes at **q** = 0 and nonlinear dispersion of acoustic modes nearby.

*Euphonic* optionally applies a correction using projection methods onto the pure translation modes to restore near-exact satisfaction of the sum rule. Two alternative adjustments are implemented: either to the dynamical matrices (the *reciprocal space* method) or to the periodic representation of the force constant matrix (the *real space* method). If equation (4*b*) is imperfectly satisfied, the dynamical matrix *D* (dropping indices for clarity) has three near-zero eigenvalues corresponding to the acoustic modes. A correction is applied, 

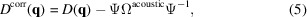

where Ψ is the matrix of eigenvectors of *D*(**q** = 0). Ω_acoustic_ is a matrix whose diagonal entries are the eigenvalues of *D*(**q** = 0) for the acoustic modes and zero otherwise. As per Gonze & Lee (1997) ▸, the correction at **q** = 0 is also applied at non-zero **q**. The alternative real space correction is similarly formulated, but applied to the force constant matrix: 

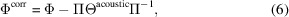

where Φ, Π and Θ^acoustic^ are all 3*NN*_cell_ × 3*NN*_cell_ matrices. Φ is the matrix of force constants, Π is the matrix of eigenvectors of Φ, and Θ^acoustic^ is a matrix whose diagonal entries are the eigenvalues of Φ for the acoustic modes and zero otherwise. This method more faithfully restores the linearity of the acoustic branches near **q** = 0 as well as the Γ-point limit.

#### 2.1.3. Polar crystals

For non-polar crystals, the decay of force constants with distance by a high inverse power law of 5–7 means that a cutoff radius of 8–10 Å is usually sufficient to contain the non-negligible elements of the force constant matrix. The resulting supercell of 16–20 Å in each dimension is well within the typical computational capability of *ab initio* density functional theory. [Typically these would be within the local-density approximation or generalized-gradient approximation, but hybrid functionals have become more accessible even for a system such as La_2_Zr_2_O_7_ (Chernyshev, 2019 ▸).] However, in the case of ionic or polar crystals the Coulomb interactions add a tail that decays as *R* ^−3^, which precludes a converged calculation in a computationally practical supercell. This long-range tail is responsible for the phenomenon of splitting of the longitudinal optical and transverse optical modes (LO/TO splitting) and unwarranted truncation will lead to unphysical behaviour of LO modes at the origin. Fortunately this term may be computed analytically and subtracted from the force constant matrix leaving the remainder term, which can be represented within a feasibly sized supercell (Gonze *et al.*, 1994 ▸). This is the approach adopted by *CASTEP* and several other codes, which output only the short-ranged part of the force constant matrix. *Euphonic* computes the dipole–dipole correction term of Gonze *et al.* (1994 ▸) using the Born effective charges and dielectric permittivity to reconstruct the full force constant matrix.

### 2.2. Coherent inelastic neutron scattering

The coherent one-phonon scattering structure factors can be calculated at the momentum transfer **Q** of scattered neutrons (Dove, 2003 ▸; Squires, 1996 ▸): 

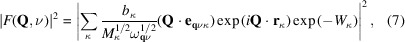

where **q** is the reduced wavevector in the first Brillouin zone (*i.e.* **Q** = **q** + **τ**, where **τ** is a reciprocal lattice vector), *b*_κ_ is the coherent neutron scattering length of atom κ, **r**_κ_ is the vector from the origin to atom κ within the unit cell, *M*_κ_ is the mass of atom κ, 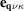 is the phonon polarization vector at **q** of mode ν for atom κ and ω_**q**ν_ is the phonon frequency of mode ν at **q**. The term exp(−*W*_κ_) is the anisotropic Debye–Waller factor for atom κ, where the exponents can be written as

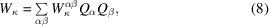




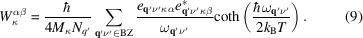

The sum in (9) is over wavevectors and modes in the first Brillouin zone (BZ), 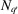 is the number of **q**-points in the sum, *T* is the temperature, α and β run over the Cartesian directions, 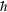 is the reduced Planck constant, and *k*_B_ is the Boltzmann constant. The Debye–Waller factor has been written in this form to make it explicit that the expensive computation of the set of 3 × 3 matrices 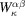 need only be performed once over an appropriately fine grid in the first Brillouin zone, leaving computation of the Debye–Waller factor for an arbitrary **Q** as the fast evaluation of a quadratic form for each atom, equation (8).

From the one-phonon structure factors the neutron dynamical structure factor *S*(**Q**, ω) can be calculated as

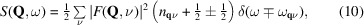

where the upper and lower signs refer to phonon creation and annihilation, respectively, and 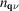 is the Bose population function 

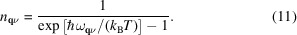

Finally, the neutron scattering cross section per unit cell in term of *S*(**Q**, ω) is 

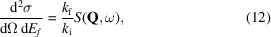

where *k*_i_ and *k*_f_ are the incident and scattered neutron wavevectors, respectively.

## 3. Implementation

**Figure 1:**
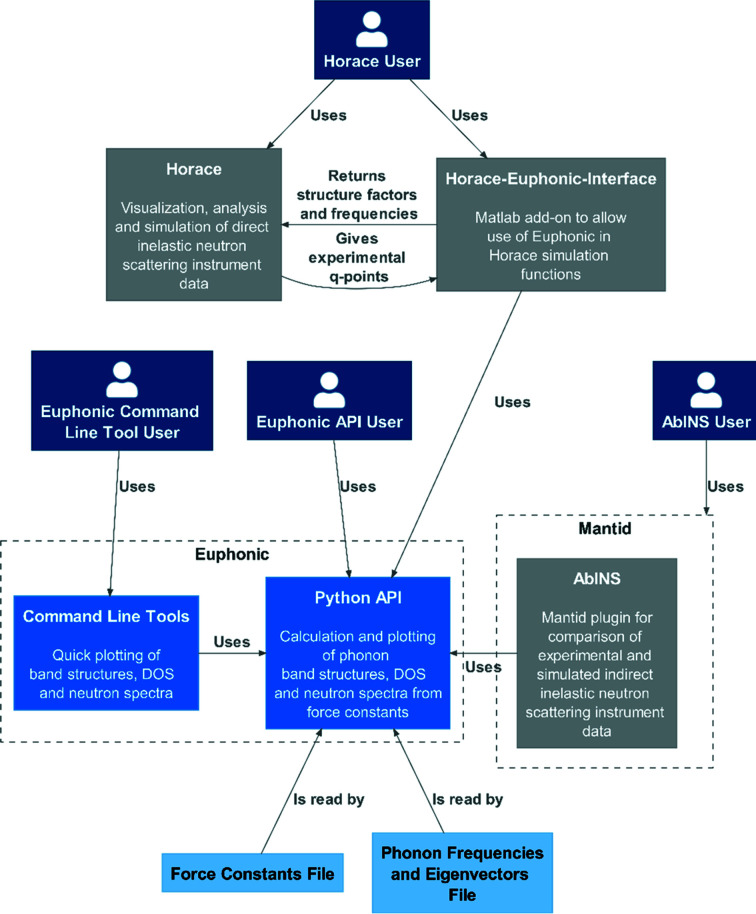
Where *Euphonic* sits in relation to other software, and users of *Euphonic* or that software.

*Euphonic* provides an extensive Python API and a number of convenient command-line tools. Fig. 1 ▸ shows how *Euphonic* connects with existing packages in the neutron software ecosystem. Established software such as *Horace* (Ewings *et al.*, 2016 ▸) and the *Mantid* (Arnold *et al.*, 2014 ▸) plug-in *AbINS* (Dymkowski *et al.*, 2018 ▸) make direct use of *Euphonic* as a calculator for simulated phonons and scattering intensities. The command-line tools provide convenient plotting of phonon band structures, DOS and the neutron dynamical structure factor along specific reciprocal lattice directions (see Section 6.1). *Euphonic* can also be used directly from Python environments for customized plots, workflows or functionality development.

### 3.1. Context and API

In a typical *Horace* workflow, the scattering intensities at millions of **Q**-points can be combined to produce multidimensional plots of measured data. Prior to the availability of *Euphonic*, the workflow for generating such plots was limited by the need to read precomputed phonon frequencies and eigenvectors from files produced by other programs. The bottleneck here becomes the activity of reading and writing to disk. For a system of *N* atoms per unit cell, each **Q**-point has a set of eigenvectors whose storage requirements of 18*N* ^2^ floating-point numbers can become impractically large. For example, a 22-atom unit cell with a modest 25 000 **Q**-points would equate to a 5 GB text file in *CASTEP* .phonon format. In a typical cluster environment running the *CASTEP* phonons tool, more than 85% of run time is spent writing this file to disk (see Table 1 ▸ for specific *N* ^2^ timing examples). Furthermore, on a shared cluster resource this can be exacerbated by local network capacity and the activities of other users.

**Figure 2:**
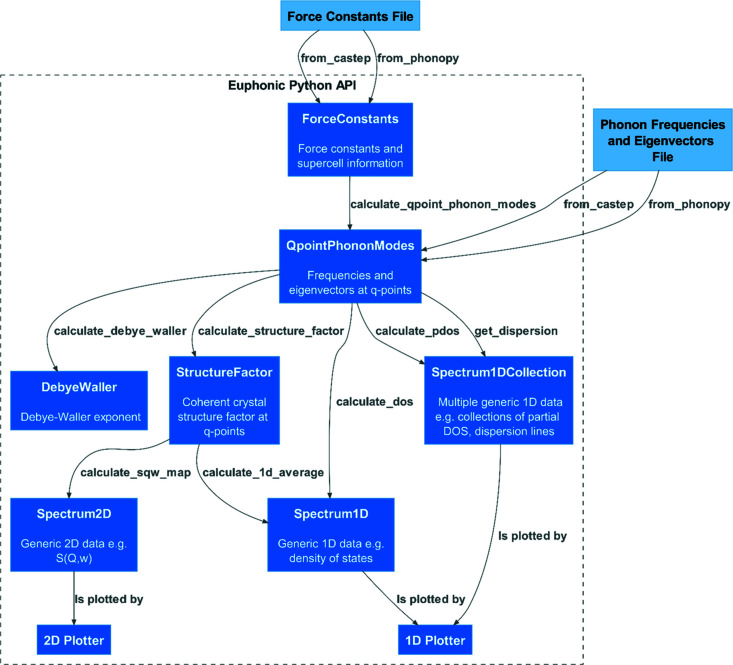
Summary of the API to *Euphonic*, showing the main classes and methods.

The requirement of efficiency for *Euphonic* has driven the decision to implement Fourier interpolation of phonon frequencies and eigenvectors directly from force constants. This allows for calculation of data for each **Q**-point on demand, removing any need for file-based data transfer from other codes. Accordingly, the representation of force constants as a class and associated methods forms the core of the *Euphonic* API, illustrated in Fig. 2 ▸ and described for reference below.

ForceConstants objects can be instantiated from Python data objects such as *Numpy* (Harris *et al.*, 2020 ▸) arrays, but would be typically created from the data outputs of an external modelling code; currently *CASTEP* .castep_bin output and *Phonopy* phonopy.yaml, FORCE_CONSTANTS and force_constants.hdf5 output are supported. Through *Phonopy*, *Euphonic* force constants can be obtained using a wide variety of atomistic codes such as *VASP* (Kresse & Furthmüller, 1996 ▸), *Abinit* (Gonze *et al.*, 2020 ▸) and *Quantum Espresso* (Giannozzi *et al.*, 2009 ▸), making *Euphonic* accessible to a large portion of the materials modelling community. From the force constants, phonon frequencies and eigenvectors may be calculated at arbitrary **Q**-points using the methods described in Section 2.1.

QpointPhononModes represents phonon mode data: the **Q**-points, phonon frequencies and eigenvectors. QpointPhononModes can also be instantiated from external datafiles (currently, these are *CASTEP* .phonon and *Phonopy* mesh/band/qpoints files). The *Pint* Python library (Grecco, 2012 ▸) is extensively used in *Euphonic* to represent dimensioned data as a Quantity with both magnitude and units. This makes the units explicit, and facilitates conversion to the preferred units of the end user (usually meV, cm^−1^ or THz) in the case of phonon frequencies. No particular **Q**-point sampling is enforced; while, for example, a Monkhorst–Pack mesh is recommended for traditional DOS plotting, phonon modes can also be calculated along a high-symmetry path or sampled at random points depending on the use case. From the phonon modes, *Euphonic* can compute quantities such as the mode-resolved structure factors, the Debye–Waller exponent, and total, partial and neutron-weighted DOS.

StructureFactor is derived from the phonon modes. This object contains the neutron structure factors resolved by the **Q**-point and phonon mode index (ν) and also the **Q**-points and frequencies. This can be binned in energy to produce a 2D *S*(**Q**, ω) plot, or averaged over the contained **Q**-points to produce a 1D *S*(ω) spectrum.

DebyeWaller represents the temperature-dependent Debye–Waller factor; specifically, it contains the set of 3 × 3 matrices, one per atom, 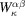 defined in equation (9). A DebyeWaller object is typically precomputed over a uniform **Q**-point mesh and then applied during a structure factor calculation to perform the fast calculation of the atomic Debye–Waller exponent *W*_κ_ via equation (8) over whichever (potentially large) set of **Q**-points are appropriate for the task at hand.

Spectrum2D, Spectrum1D and Spectrum1DCollection are classes representing generic spectrum objects that can be used for various purposes, such as representing the DOS with Spectrum1D, or an *S*(**Q**, ω) or *S*(|**Q**|, ω) intensity map with Spectrum2D. Band structure and partial DOS data are represented with Spectrum1DCollection, which ensures that consistent bins are used and allows individual lines to be tagged with metadata. The plotting tools in turn work with the generic spectrum objects to produce plots of phonon band structure, DOS and intensity maps.

Crystal is a simple class containing the crystal structure information: the cell vectors, atom positions, species and masses. The above ForceConstants, QpointPhononModes, StructureFactor and DebyeWaller classes all contain an instance of this crystal class as an attribute, to ensure that the data in each class remain complete and unambiguous.

### 3.2. Use with *Horace*

A widely used software application for analysis and visualization of multidimensional TOF INS data from single-crystal experiments is *Horace* (Ewings *et al.*, 2016 ▸). In addition to handling and plotting the data, it also allows simulation and fitting of these data sets with user-created models of the scattering function. A MATLAB add-on has been developed, *Horace-Euphonic-Interface* (Fair & Le, 2022 ▸), which provides interface functions that allow *Euphonic* to be used to simulate data sets directly in *Horace*. The way this works is illustrated in Fig. 1 ▸. First a user sets up a model with *Horace-Euphonic-Interface*, providing the path to the force constants file or folder, and adding other optional parameters such as the sample temperature or the Debye–Waller grid size. The user then calls a *Horace* simulation function with the data set to be simulated and the model they have just created. *Horace* will automatically provide the **Q**-points to be simulated to *Horace-Euphonic-Interface*, which then calls *Euphonic* to calculate the structure factors and phonon frequencies at those **Q**-points. *Horace-Euphonic-Interface* then converts the output from *Euphonic* into the required form for *Horace* to create the simulated data set.

This is a significant improvement over previous workflows to simulate scattering from phonons, which required users to program their own functions to calculate the structure factors from *ab initio* calculations. These would not usually include Fourier interpolation of the force constants, and hence were restricted to the **Q**-points output by the *ab initio* calculations. These bespoke scripts were also not typically optimized for fast computation. *Horace-Euphonic-Interface* has allowed users to easily simulate on exactly the same axes and in the same software as the experimental data, making fitting of scaling factors and quantitative comparisons much more convenient. The performance of *Euphonic* has also made application of the Monte Carlo based instrumental resolution convolution method (Perring, 1991 ▸) available in *Horace* tractable with phonons for the first time. Examples of data simulated and fitted with *Euphonic* and *Horace* are shown in Section 6.3. *Horace-Euphonic-Interface* is open source and distributed as a MATLAB Toolbox file on Github and the MATLAB File Exchange. Links to the source code and online documentation are available at https://doi.org/10.5286/SOFTWARE/HORACEEUPHONICINTERFACE (Fair & Le, 2022 ▸).

### 3.3. Use with *AbINS*

*AbINS* is a code which simulates powder-averaged INS spectra in an analytic incoherent approximation, and resides in the *Mantid* framework used for experimental data reduction and analysis (Dymkowski *et al.*, 2018 ▸; Arnold *et al.*, 2014 ▸; Akeroyd *et al.*, 2013 ▸). *AbINS* has recently been updated to make use of *Euphonic*; as of *Mantid* version 6.3, it is possible to select force constants data in *CASTEP* or *Phonopy* format as an input file. End users do not need to know anything about *Euphonic* or change their workflow, except to ensure that force constants data are present in their .castep_bin or phonopy.yaml files. A single-parameter cutoff distance [as defined by Moreno & Soler (1992) ▸, and hidden from the user interface] is used to determine a default Monkhorst–Pack mesh for the given crystal structure; eigenvalues and eigenvectors are computed using the *Euphonic* Python API and passed on to the usual incoherent inelastic scattering computation (Dymkowski *et al.*, 2018 ▸). *AbINS* and *Horace* have different target users: with *Euphonic* as a common dependency, it becomes possible for these neutron scattering simulation codes to develop overlapping feature sets while sharing implementation work.

## 4. Performance profiling and optimization

Given the aim of calculating scattering intensities at millions of **Q**-points, performance optimization of *Euphonic* has been a priority. Table 1 ▸ illustrates the potential cost of writing large eigenvector arrays to disk, which has been avoided in *Euphonic* by enabling its own interpolation from force constants. There are four main steps in computing the neutron dynamical structure factor from force constants: reading the force constants, computing the phonon frequencies and eigenvectors (interpolation), computing the mode-resolved structure factors, and, finally, applying the Bose factor and binning these in energy to obtain *S*(**Q**, ω). Reading the force constants only has to be done once, and the binning for large data sets is typically done via another program such as *Horace*.

Of the two remaining steps, the calculation of phonon frequencies and eigenvectors is by far the most expensive, illustrated in Table 2 ▸. Even in the case of La_2_Zr_2_O_7_, which has the most expensive structure factor calculation due to the number of atoms in the unit cell, the interpolation takes approximately 20 times longer. For this reason, this part of the calculation has been the focus of much of the optimization effort, and performance comparisons have been made with another interpolation tool, the *CASTEP* phonons tool, rather than software that performs the cheaper structure factor calculations such as *ab2tds* (Mirone & Wehinger, 2013 ▸) or *OClimax* (Cheng *et al.*, 2019 ▸; Cheng & Ramirez-Cuesta, 2020 ▸). The *Phonopy* software does perform phonon interpolation but has not been chosen for performance comparisons, as it does not (as of version 2.11.0) parallelize its calculation over **Q**-points.

**Figure 3:**
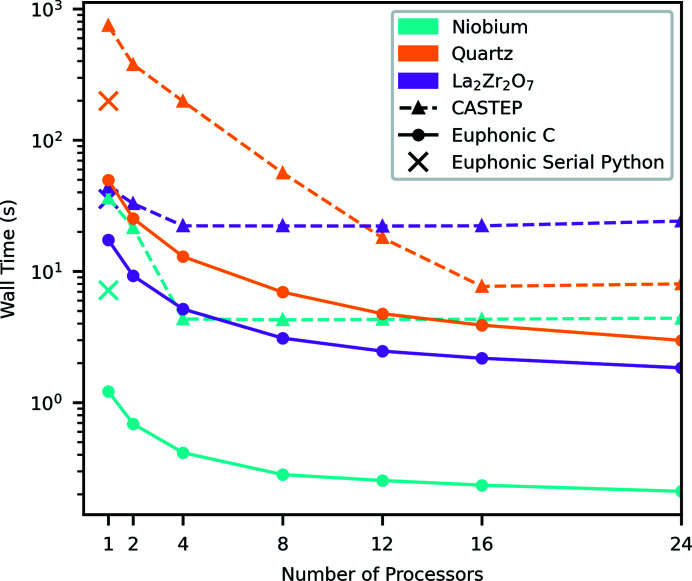
Comparison of the wall time taken to run the calculate_qpoint_phonon_modes interpolation function in *Euphonic* against the phonon_calculate function in *CASTEP* for 25 000 **Q**-points for different materials and numbers of processors. Scatter points show the wall time taken to run the serial Python version of calculate_qpoint_phonon_modes. Details of the hardware are given in Section 4.1.

*Euphonic* makes extensive use of *Numpy* to improve its performance, but the serial Python performance shown in Table 2 ▸ is still not sufficient to simulate the number of **Q**-points contained in larger multidimensional **Q**–ω data sets in a reasonable time. Accordingly, an extension has been written in C and OpenMP to perform the interpolation part of the calculation, which both improves performance significantly and enables calculations to be carried out in parallel. The performance improvement can be seen in Fig. 3 ▸, which shows the time taken to run the calculate_qpoint_phonon_modes interpolation function in *Euphonic* for 25 000 **Q**-points for different materials, for both the serial Python and the parallel C implementations. One metric for comparing performance is the speedup calculated as the ratio of the times to perform the same operation in the two implementations: 

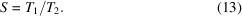

For one processor, use of the C extension provides speedups of 2.1, 4.0 and 6.0 over the pure Python implementation for La_2_Zr_2_O_7_, quartz and Nb, respectively. Fig. 3 ▸ also shows how the wall time changes with increasing numbers of processors and compares it with the time taken to run the phonon_calculate function from the *CASTEP* phonons tool. Care has been taken to make a fair comparison, so *CASTEP* features that are not available in *Euphonic* which would have decreased the performance of *CASTEP* have been switched off (group theory analysis, dynamical matrix symmetrization) and features that could not be turned off have been profiled and subtracted from the total time (writing the .phonon file, constructing the force constant matrix). Even with this taken into account, the performance of *Euphonic* is better than that of the *CASTEP* phonons tool by an order of magnitude in some cases, with *Euphonic* giving speedups of 13.1, 2.7 and 20.9 over the *CASTEP* tool for La_2_Zr_2_O_7_, quartz and Nb, respectively, for 24 processors.

It can be seen in Fig. 3 ▸ that interpolation for quartz is slower than for La_2_Zr_2_O_7_, despite the former having fewer atoms per unit cell. This is due to the expensive Ewald sum correction that must be applied to the dynamical matrix for polar materials, described in Section 2.1. This calculation has been heavily optimized in *Euphonic*, which explains the performance difference between *Euphonic* and the *CASTEP* phonons tool for quartz. In the cases of Nb and La_2_Zr_2_O_7_, the performance discrepancy largely comes from the fact that the *CASTEP* phonons tool uses distributed memory parallelism via MPI, so needs to communicate the phonon frequencies and eigenvectors back to the main process, which is where most time is spent. *Euphonic* does not have this issue as it makes use of shared memory parallelism via OpenMP. This was chosen specifically to satisfy the two main-use cases for *Euphonic*: running smaller calculations on a single computer or node; and running larger calculations on a cluster via a data analysis tool such as *Horace*, in which case *Horace* would handle any multi-node parallelism.

**Figure 4:**
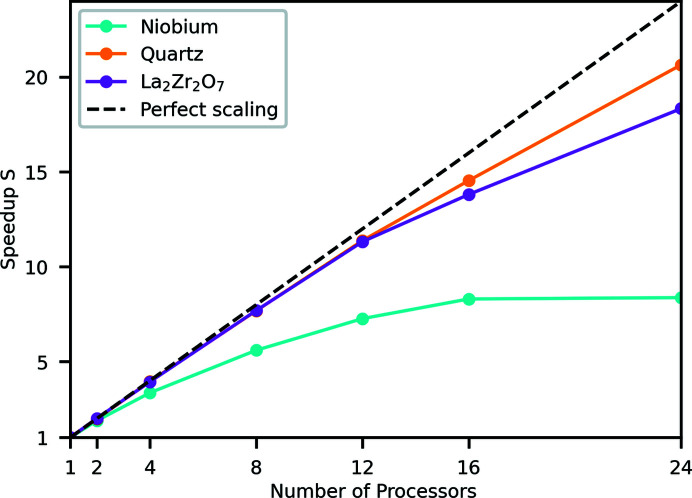
Speedup of the calculate_qpoint_phonon_modes interpolation function in *Euphonic* with C extension compared with serial Python for 250 000 **Q**-points for different materials and numbers of processors. Details of the hardware are given in Section 4.1.

**Figure 5:**
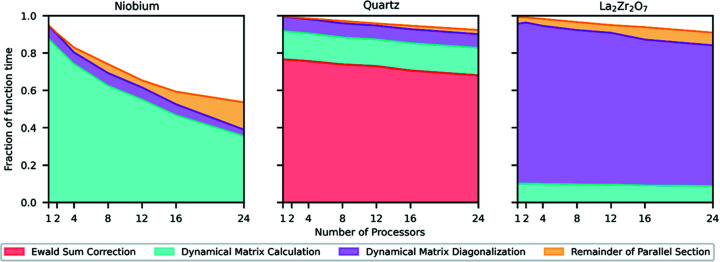
Where time is spent in the *Euphonic* interpolation function calculate_qpoint_phonon_modes for 250 000 **Q**-points for different materials and numbers of processors. The white areas indicate time spent in the serial part of the code.

The number of **Q**-points used for comparison of *Euphonic* with other tools, 25 000 for the benchmarking presented above, was chosen because it pushes the limits of the *CASTEP* tool, and allows results to be obtained in a reasonable amount of time. However, the run times of just a few seconds for *Euphonic* for large numbers of processors are not enough to obtain good performance data, as a non-negligible amount of time will be spent in non-computational parts of the code, for example importing libraries or spawning threads. The interpolation in *Euphonic* has therefore also been profiled for 250 000 **Q**-points, and has been used to demonstrate the performance scaling; this is shown in Fig. 4 ▸. The speedup has been calculated as in equation (13), where *T*_1_ is the serial interpolation function time, and *T*_2_ is the parallel time. Each interpolated **Q**-point is independent, so the calculation can easily be parallelized over **Q**-points using a parallel for loop. Despite this independence of **Q**-points, the scaling is imperfect, particularly for Nb. This can be explained by looking at Fig. 5 ▸, which shows the time spent in different parts of the interpolation calculation in C for different numbers of processors and materials. In particular, the serial Python part of the calculation shown in white imposes a ∼0.1 s overhead, which limits the maximum possible speedup, especially for a small system like Nb where the parallelized part only takes 0.1 s with 24 processors. The serial part includes various setup tasks, such as calculating the list of periodic supercell images as described in Section 2.1.1.

Fig. 5 ▸ also explains the longer run times for quartz observed, shown in Fig. 3 ▸. Even after being the focus of much optimization work, the Ewald sum still takes around 70% of the total interpolation time for quartz. This optimization has included avoiding redundant calculation of **Q**-independent values by factorization, and optimizing the balance between the real and reciprocal space sums [Λ in equation (5) of Gonze *et al.* (1994) ▸]. Changing Λ will not change the result but can drastically improve the performance if Λ is chosen correctly. The optimum value depends on the material and is not immediately obvious. *Euphonic* provides a command-line tool, euphonic-optimise-dipole-parameter, which profiles the calculation for a few **Q**-points and suggests the optimum value for that system for use in further calculations. Even with these optimizations, there is still a performance penalty for the calculation of phonons for polar materials, suggesting a target for further optimization work. In non-polar materials, most of the time is spent either calculating or diagonalizing the dynamical matrix. This depends strongly on the number of atoms in the unit cell versus the number of cells in the supercell. In the case of La_2_Zr_2_O_7_, which has 22 atoms in the unit cell, over 70% of the interpolation time is spent diagonalizing the dynamical matrices. By contrast Nb has 1 atom per unit cell and (ignoring serial overhead) over 80% of the time is spent calculating dynamical matrices.

### 4.1. Hardware and software libraries

All profiling in this section has been completed on the STFC Scientific Computing Department’s SCARF Cluster using the SCARF 18 hardware, which contains 2 Intel Gold 6126 processors per node (24 cores per node). *Euphonic 0.6.1* and *CASTEP 19.1* were used. Both the *Euphonic* C extension and the *CASTEP* phonons tool have been compiled with the Intel 2018.3 compiler, OpenMPI 3.1.1 and linked against Intel MKL 2018.3. The profiling results can be obtained at https://github.com/pace-neutrons/euphonic-performance.

## 5. Validation

The results of a comparison of *Euphonic* output with experimental INS data will be given in Section 6.3. In this section, to test the calculation of the dynamical structure factor stringently, *Euphonic* is validated against two other programs: *ab2tds* (Mirone & Wehinger, 2013 ▸) and *OClimax* (Cheng *et al.*, 2019 ▸; Cheng & Ramirez-Cuesta, 2020 ▸). This allows validation of the more subtle parts of the calculation (such as the Debye–Waller factor) without the complication of other scattering mechanisms or broadening due to instrumental resolution, or the possibility of the first-principles computation of the force constant matrix failing to fully capture the physics of the lattice dynamics. For validation comparisons, four materials have been chosen: La_2_Zr_2_O_7_, quartz, Nb and Al. La_2_Zr_2_O_7_, quartz and Nb force constants, frequencies and eigenvectors have been computed using *CASTEP*, and the corresponding data for Al have been computed using *VASP* and *Phonopy*, to validate both *CASTEP* and *Phonopy* readers available in *Euphonic*.

**Figure 6:**
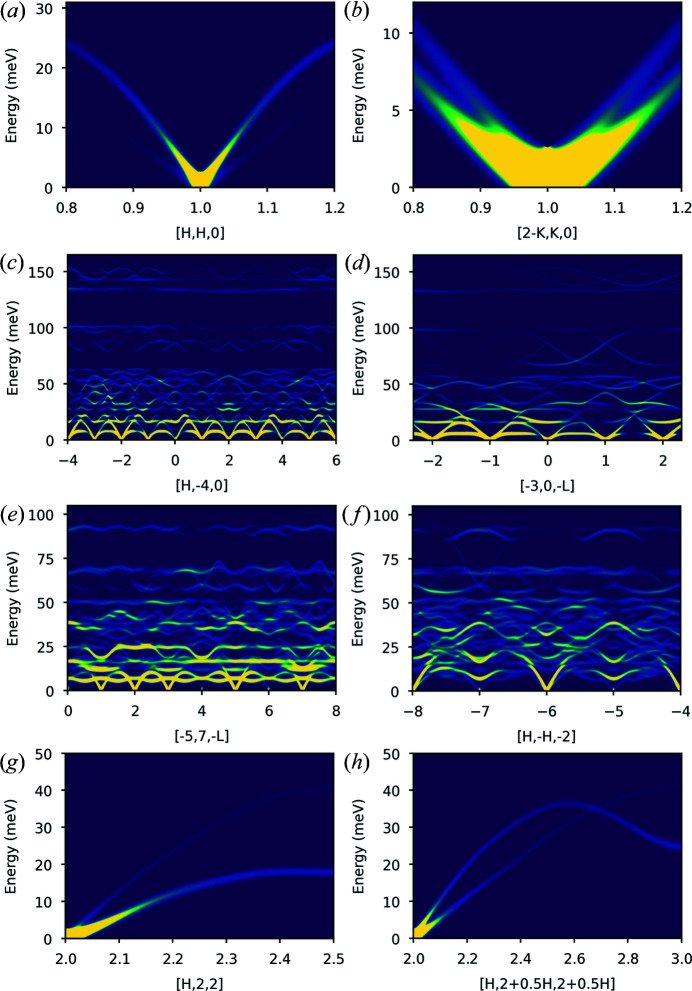
Neutron dynamical structure factors for the validation cuts simulated with *Euphonic*. Nb along (*a*) [*h*, *h*, 0] and (*b*) [2 − *k*, *k*, 0]. Quartz along (*c*) [*h*, −4, 0] and (*d*) [−3, 0, −1]. La_2_Zr_2_O_7_ along (*e*) [−5, 7, −1] and (*f*) [*h*, −*h*, 2]. Al along (*g*) [*h*, 2, 2] and (*h*) [*h*, 2 + *h*/2, 2 + *h*/2].

The data chosen for validation are all 2D (**Q**, ω) maps as these types of data can be calculated by all three programs. The data maps have a wide variation of magnitudes and directions in **Q** to ensure the variation of quantities such as structure factors and Debye–Waller factors across **Q** space are reliably tested. Visualizations of the chosen (**Q**, ω) maps are shown in Fig. 6 ▸. The metric that has been used for comparison is the mean relative percentage difference (MRPD): 

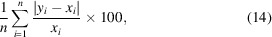

where *x_i_* is the neutron dynamical structure factor [*S*(**Q**, ω) as in equation (10)] calculated with *Euphonic*, and *y_i_* is the equivalent calculated with *OClimax* or *ab2tds*. Acoustic modes close to the gamma point, which have diverging intensity, and very low intensity data have been excluded to avoid numerical instabilities (more details are given in the supporting information). The *OClimax* neutron dynamical structure factors have been read directly from *OClimax* output. In the case of *ab2tds*, the instrumental resolution applied when creating such maps could not be completely removed from the *ab2tds* output, so the dynamical structure factor has instead been calculated by binning in energy the *ab2tds* mode-resolved output [which is equivalent to the one-phonon structure factor in equation (7) with the (*n*_**qν**_ + 1) factor from equation (10) already applied].

The results of the comparisons are summarized in Tables 3 ▸ (*ab2tds*) and 4 ▸ (*OClimax*). The right-hand column of each table shows the MRPD for *S*(**Q**, ω) calculated from phonon frequencies and eigenvectors obtained from interpolation via *CASTEP* (Nb, quartz, La_2_Zr_2_O_7_) or *Phonopy* (Al). These MRPDs test the computation of *S*(**Q**, ω) by *Euphonic* directly from the frequencies and eigenvectors. The previous column shows the comparison when, in the case of *Euphonic*, *S*(**Q**, ω) is computed from the force constants, in order to test the interpolation available in *Euphonic* in addition to the *S*(**Q**, ω) calculation from the phonon frequencies and eigenvectors computed by that interpolation. For *ab2tds* the agreement is extremely good, with MRPDs of 0.05% or less. In the case of *OClimax*, the MRPDs are significantly larger, up to 2.62% for the La_2_Zr_2_O_7_ [−5, 7 −*l*] cut at 300 K. However, the overall comparison is still reasonably good, without particularly systematic variation of the relative percentage difference across the slices. The scripts used for validation and the *Euphonic*, *ab2tds* and *OClimax* inputs and outputs are available at https://github.com/pace-neutrons/euphonic-validation. Further details on the validation calculations are provided in the supporting information.

## 6. Examples

### 6.1. Command-line tools and plotting

**Figure 7:**
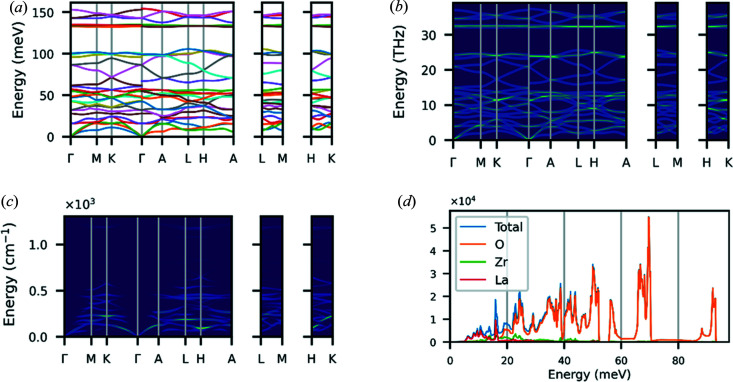
Examples of plots produced with command-line tools in *Euphonic*. (*a*) Quartz with euphonic-dispersion. (*b*) Quartz with euphonic-intensity-map using the DOS-weighted intensities option. (*c*) Quartz with euphonic-intensity-map using the neutron dynamical structure factor weighted intensities option. (*d*) La_2_Zr_2_O_7_ with euphonic-dos using the coherent-weighted partial DOS option.

This section illustrates some of the main features of *Euphonic*, using a variety of samples for both single crystals and powders from a range of modelling codes. An overview of each of the command-line tools in *Euphonic* is shown in Table 5 ▸, with example figures for each command shown in Figs. 7 ▸, 8 ▸, 10 and 11. Many of these tools allow sampling parameters (*e.g.* energy bins, broadening) and appearance options (*e.g.* unit conversions, axis labels and styling) to be easily specified via command-line arguments.

**Figure 9:**
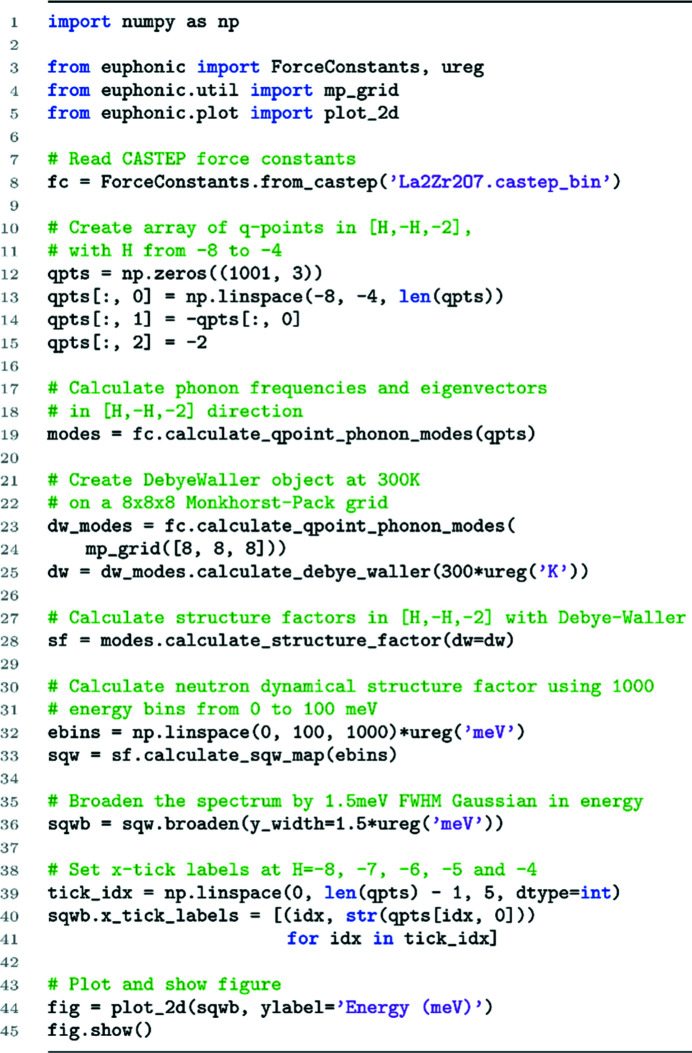
Example *Euphonic* script to calculate and plot the neutron dynamical structure factor in the [*h*, −*h*, −2] direction, producing a plot similar to Fig. 6 ▸(*f*)

For custom plots it may be necessary to write a Python script. For example, Fig. 6 ▸ shows the neutron dynamical structure factor sampled along arbitrary **Q** directions. A sample script to achieve this kind of result is shown in Fig. 9 ▸.

### 6.2. Experimental powder data comparison

**Figure 10:**
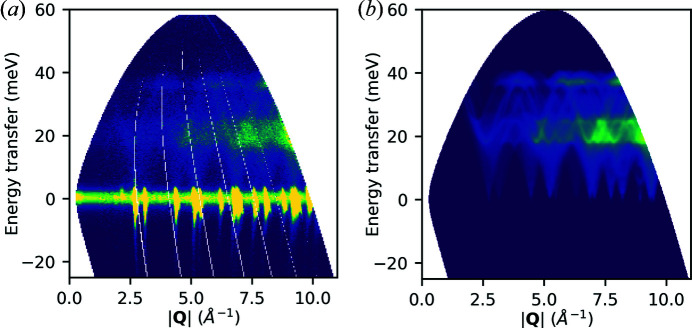
Al powder data at 5 K. (*a*) Experimental data recorded on MARI. (*b*) Corresponding powder-averaged coherent *S*(|**Q**|, ω) generated with euphonic-powder-map.

Here, prior experimental measurements are compared with newly simulated spectra (Fair *et al.*, 2022*b* ▸). Measurements of powdered elemental samples of Al (at 5 K with 60 meV incident energy and the Gd monochromating chopper running at 200 Hz) and Si (at 300 K with 80 meV incident energy with the ‘sloppy’^1^ monochromating chopper at 250 Hz) were recorded on the MARI instrument at ISIS. The data were reduced using *Mantid* and binned to 2D *S*(|**Q**|, ω) maps using *MSLICE* in *Mantid* [Figs 10(*a*) ▸ and 11(*a*) ▸]. For the Al calculations, *VASP* (version 5.4.4) was used, and the force constants were obtained by finite displacements using *Phonopy* (Kresse & Furthmüller, 1996 ▸; Kresse & Joubert, 1999 ▸; Togo & Tanaka, 2015 ▸). For Si, *CASTEP* was used, obtaining force constants by density-functional perturbation theory (Clark *et al.*, 2005 ▸; Refson *et al.*, 2006 ▸). For more details, see the supporting information.

**Figure 11:**
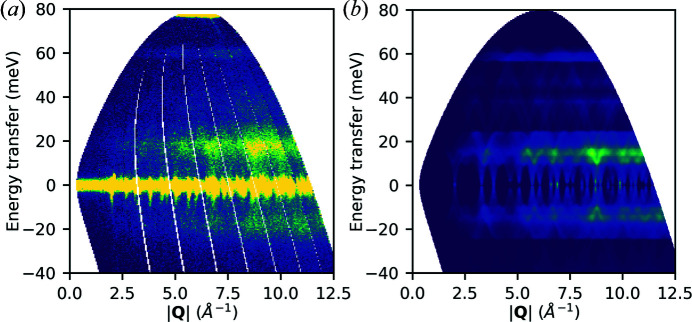
Si powder data at 300 K. (*a*) Experimental data recorded on MARI. (*b*) Corresponding powder-averaged coherent *S*(|**Q**|, ω) generated with euphonic-powder-map.

The resulting castep.bin and phonopy.yaml files were used with the euphonic-powder-map command-line tool to generate numerically sampled maps of *S*_coh_(|**Q**|, ω). Details of the parameters are given in the supporting information. These results are plotted in Figs. 10(*b*) ▸ and 11(*b*) ▸ and include the main inelastic scattering features in the positive region of the experimental measurements. It is easy to see in both the experimental and the simulated data how spherically averaged periodic features collide to create broad regions of higher intensity. In the case of the 300 K Si data, both the experimental and the simulated results have significant intensity in the negative energy transfer region, whereas for the 5 K Al data the intensity is suppressed in this region. This arises from the Bose population factor suppressing the low-temperature scattering. The high intensities seen around zero energy transfer in the experimental measurements in Figs. 10(*a*) ▸ and 11(*a*) ▸ are attributed to scattering from Bragg peaks which is not currently modelled by *Euphonic*, hence they are absent from the corresponding simulations in Figs. 10(*b*) ▸ and 11(*b*) ▸. The high-intensity feature in Fig. 11(*a*) ▸ at 80 meV is an artefact due to a later pulse of neutrons which passes through the chopper system, and is not due to scattering from the sample, so is not reproduced in the simulated data in Fig. 11(*b*) ▸.

### 6.3. Experimental single-crystal data comparison using *Horace*

Simulations created from preexisting quartz force constant calculations have been compared with newly collected experimental data (Fair *et al.*, 2022*b* ▸). Original quartz force constant calculations performed with *CASTEP 6.1* have been re-processed for this work with *CASTEP 19.1*; more details are provided in the supporting information. For the experimental measurements, a large single crystal of natural quartz was aligned with the *c* axis vertical on an Al plate, secured in place with Al wire. It was cooled to 10 K using a closed-cycle refrigerator. The MERLIN ‘G’ chopper at 350 Hz was phased to select 45 meV incident energy neutrons. The sample was rotated over 180° in 0.5° steps. The data for each individual angle were reduced using *Mantid* and then combined using *Horace*.

**Figure 12:**
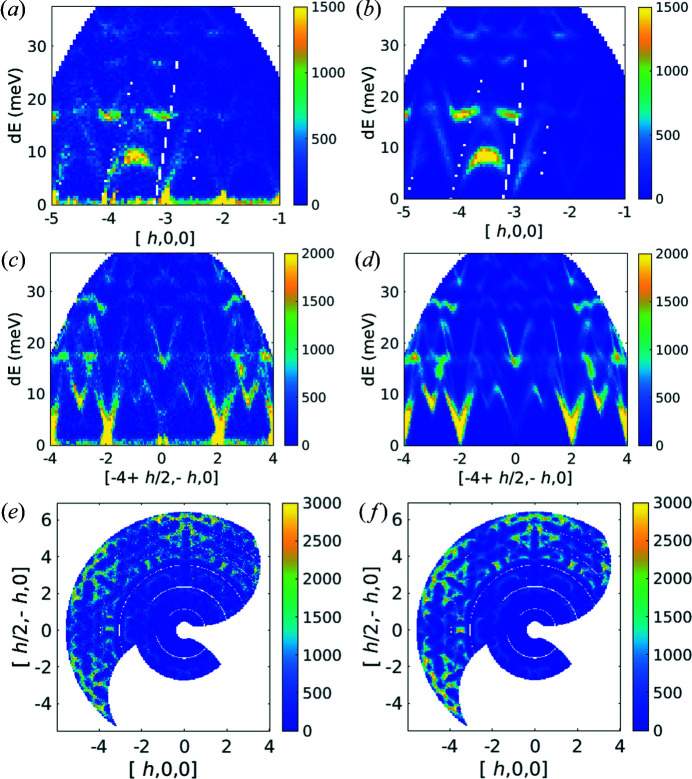
(*a*), (*c*) and (*e*) Cuts through the experimental quartz data set and (*b*), (*d*) and (*f*) corresponding simulations with *Euphonic*. All figures use the projection axes of [*h*, 0, 0], [*h*/2, −*h*, 0], [0, 0, 1] with integration over the non-plotted directions of ±0.1. The energy axis integration in (*e*) and (*f*) is from 8 to 9 meV.

**Figure 13:**
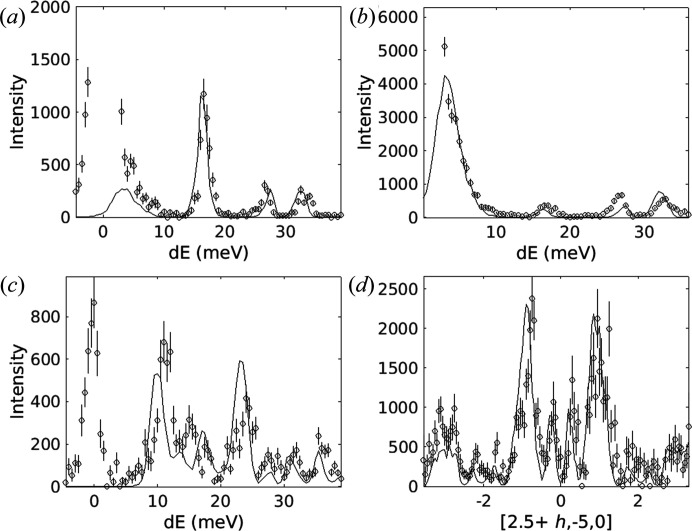
Linecuts through the quartz data and corresponding simulations with *Euphonic* (solid lines). A cut through (*a*) [−3, 0, 0], (*b*) [−5, 1, 0], (*c*) [−3.75, −0.5, 0] and (*d*) a cut at constant energy (integrated from 8 to 9 meV). All figures use the projection axes [*h*, 0, 0], [*h*/2, −*h*, 0] and [0, 0, 1] with integration over the non-plotted directions of ±0.1.

Several cuts through experimental and simulated data for quartz are shown in Fig. 12 ▸. The neutron dynamical structure factor has been computed with *Euphonic*, with the instrumental resolution accounted for by the Monte Carlo resolution convolution method (Perring, 1991 ▸) implemented in *Horace*. In addition, 1D cuts are shown in Fig. 13 ▸, with the simulated scattering likewise convolved with the instrumental resolution function. The only adjustable parameter in the comparison is a global scaling factor which has been determined by setting the integrated areas of the experimental and simulated data in Fig. 13 ▸(*a*) between 12 and 37 meV to be equal, which has then been applied to all 1D and 2D cuts. Aside from this (arbitrary) choice of intensity scale, no adjustable parameters have been used in any part of the calculation. There are small disagreements; for example, the phonon frequencies do not match up perfectly. This is to be expected, as the local density approximation tends to overestimate bond strengths (and thus phonon frequencies). In addition there are small differences in particular mode intensities, for example around 27 meV in Fig. 13 ▸(*b*) where the intensity is underestimated, or at 23 meV in Fig. 13 ▸(*c*) where it is overestimated. However, given the lack of adjustable parameters the agreement is remarkably strong. Notwithstanding, disagreements that arise from the *ab initio* code not fully capturing the physics of the material [Figs. 13 ▸(*a*)–13(*d*)] show the importance of accounting for instrumental resolution when comparing calculation with experimental data.

## 7. Conclusions and future development

We have described the *Euphonic* package, which is designed to efficiently compute phonon eigenvectors, eigenvalues and the INS cross section for a large number of **Q**-points from force constant matrices. It has a set of command-line tools to plot the phonon band structure, DOS and neutron dynamical structure factor along a path in reciprocal space, and an extensive Python API so that it can be used directly from Python environments for customized workflows and plotting. *Euphonic* also works directly from *Horace* (Ewings *et al.*, 2016 ▸) and the *Mantid* (Arnold *et al.*, 2014 ▸) plug-in *AbINS* (Dymkowski *et al.*, 2018 ▸).

Examples of the use of the *Euphonic* command-line tools were shown in Section 6, together with a comparison of simulated and experimental data from powders and single crystals. The latter also includes convolution with the instrumental resolution function.

*Euphonic* has been extensively benchmarked and validated against other codes. It is now being used for the interpretation of phonon data at the ISIS Neutron and Muon Source, where it will continue to be maintained and developed as part of the core data analysis software portfolio. Promising avenues for future development include support of other codes, particularly the *Atomic Simulation Environment* (*ASE*) (Hjorth Larsen *et al.*, 2017 ▸); performance improvements from symmetry-aware interpolation of the dynamical matrices and other interpolation methods; corrections for thermal expansion/softening using a quasi-harmonic force constant matrix; and X-ray structure factors.

*Euphonic* is open source and the source code is available to download from Github (Fair *et al.*, 2022*a* ▸), with releases also available via the *pip* and *conda* package managers, as a service to the neutron scattering and the computational chemistry and physics communities. The authors welcome bug reports, feedback on usability and documentation, and suggestions for additional functionality. The authors also welcome contributions to the *Euphonic* package.

## 8. Related literature

The following additional references are cited in the supporting information: Byrd *et al.* (1994 ▸); Francis & Payne (1990 ▸); Frank *et al.* (1995 ▸); Rappe *et al.* (1990 ▸). 

## Supplementary Material

Supporting information: Table S1 and commands. DOI: 10.1107/S1600576722009256/in5072sup2.pdf

Example Python script to calculate and plot the neutron dynamical structure factor in the [hkl] direction with Euphonic. DOI: 10.1107/S1600576722009256/in5072sup1.txt

Experimental and simulated data sets for five materials (aluminium, La2Zr2O7, niobium, quartz and silicon) for use with the Euphonic program.: https://doi.org/10.5281/zenodo.6620084

The Euphonic Python package: https://doi.org/10.5286/SOFTWARE/EUPHONIC

Associated data: https://doi.org/10.5286/ISIS.E.RB1930013

## Figures and Tables

**Figure 8:**
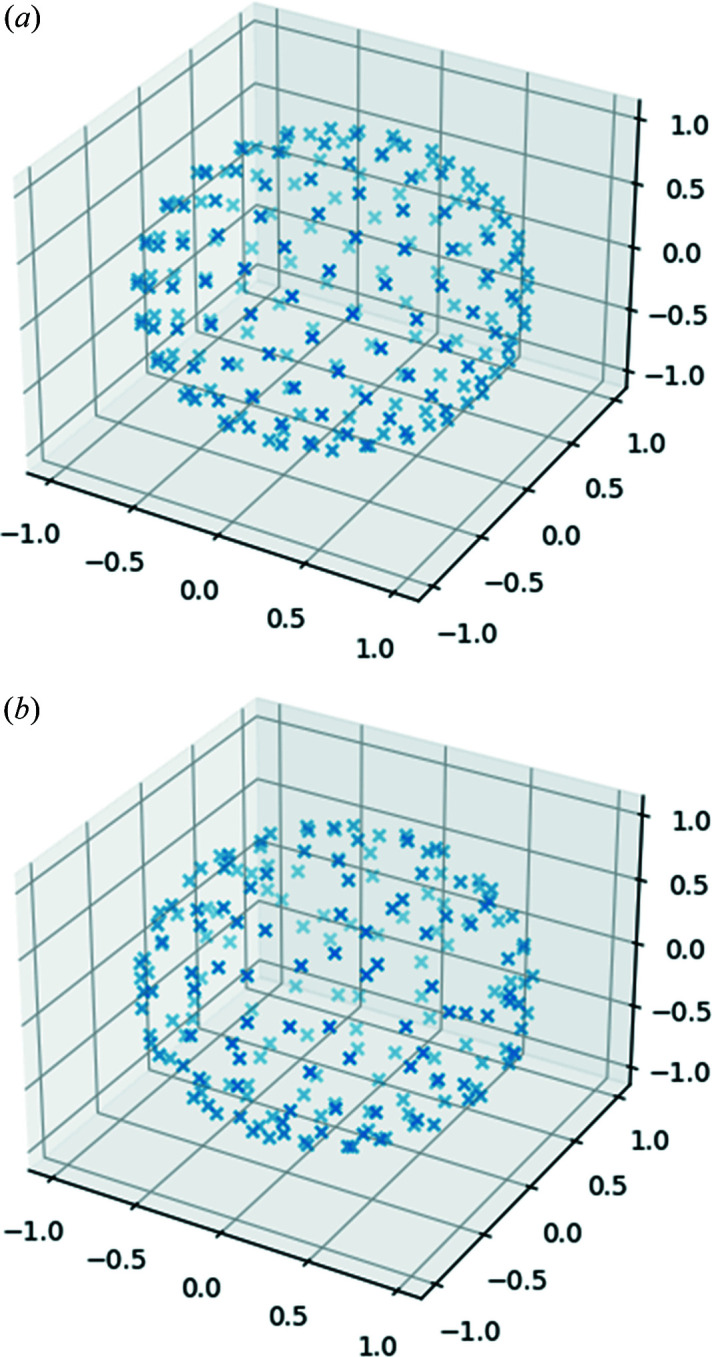
Outputs of euphonic-show-sampling. (*a*) ‘Golden sphere’ sampling. (*b*) ‘Improved spherical polar’ sampling with jitter.

**Table 1:** *CASTEP* .phonon file size and time taken to write the .phonon file compared with the total time taken to run the *CASTEP* phonons tool (including write time) with 25 000 **Q**-points for different materials Details of the hardware are given in Section 4.1.

Material	Atoms	Size (GB)	Total time (s)	Write time (s)
Nb	1	0.025	57.837	7.557
Quartz	9	0.913	811.430	64.930
La_2_Zr_2_O_7_	22	5.187	302.167	258.243

**Table 2:** Comparison of the mean time taken for phonon interpolation against the mean time taken to calculate the mode-resolved structure factors for 25 000 **Q**-points with serial Python in *Euphonic* Details of the hardware are given in Section 4.1.

Material	Interpolation (s)	Structure factor (s)
Nb	7.070	0.104
Quartz	199.069	0.426
La_2_Zr_2_O_7_	35.743	1.853

**Table 3:** Mean relative percentage differences between the *Euphonic* and *ab2tds* neutron dynamical structure factors for the Nb, quartz and La_2_Zr_2_O_7_ 2D (**Q**, ω) maps shown in Fig. 6 ▸ at 300 K For *Euphonic* the neutron dynamical structure factor has been calculated from both *CASTEP*-interpolated and *Euphonic*-interpolated frequencies and eigenvectors.

		Mean relative percentage difference	
Material	**Q** direction	*Euphonic* interpolation	*CASTEP* interpolation
Nb	[*h*, *h*, 0]	<0.01	<0.01
	[2 − *k*, *k*, 0]	<0.01	<0.01
Quartz	[*h*, −4, 0]	0.03	0.03
	[−3, 0, −1]	0.05	<0.01
La_2_Zr_2_O_7_	[−5, 7, −1]	0.05	0.05
	[*h*, −*h*, 2]	0.05	0.05

**Table 4:** Mean relative percentage differences between the *Euphonic* and *OClimax* neutron dynamical structure factors for the 2D (**Q**, ω) maps shown in Fig. 6 ▸ at different temperatures For *Euphonic* the neutron dynamical structure factor has been calculated from both *CASTEP*/*Phonopy*-interpolated [(Nb, quartz and La_2_Zr_2_O_7_)/Al] and *Euphonic*-interpolated frequencies and eigenvectors.

			Mean relative percentage difference	
Material	**Q** direction	T (K)	*Euphonic* interpolation	*CASTEP*/*Phonopy* interpolation
Nb	[*h*, *h*, 0]	300	<0.01	<0.01
		5	<0.01	<0.01
	[2 − *k*, *k*, 0]	300	<0.01	<0.01
		5	<0.01	<0.01
Quartz	[*h*, −4, 0]	300	0.87	0.87
		5	0.49	0.49
	[−3, 0, −1]	300	1.75	1.82
		5	0.83	0.92
La_2_Zr_2_O_7_	[−5, 7, −1]	300	2.62	2.62
		5	1.83	1.83
	[*h*, −*h*, 2]	300	2.05	2.05
		5	1.42	1.42
Al	[*h*, 2, 2]	300	0.01	<0.01
		5	0.01	<0.01
	[*h*, 2 + *h*/2, 2 + *h*/2]	300	<0.01	<0.01
		5	<0.01	<0.01

**Table 5:** Overview of the command-line tools available in *Euphonic*

Tool	Description	Example figure
euphonic-dispersion	Plots a phonon band structure along a recommended **Q**-point path (generated by *SeeK-path*; Hinuma *et al.*, 2017 ▸) if using a force constants file as input, or will plot existing phonon frequencies if using a phonon modes file as input (*e.g.* *CASTEP* .phonon) file.	Fig. 7(*a*)
euphonic-dos	Plots a total or partial DOS on a specified Monkhorst–Pack grid if using a force constants file, or on a precalculated grid if using a phonon modes file. The spectrum can also be weighted by coherent or incoherent neutron scattering cross section.	Fig. 7(*d*)
euphonic-intensity-map	Plots a 2D (**Q**, ω) crystal intensity map along a recommended **Q**-point path if using force constants, or along a precalculated path if using phonon modes. The intensities can be weighted by the neutron dynamical structure factor or phonon DOS.	Figs. 7(*b*) and 7(*c*)
euphonic-powder-map	Plots a 2D (|**Q**|, ω) powder intensity map along a specified range in |**Q**| using spherical averaging; requires force constants. Intensities can be weighted by the neutron dynamical structure factor or phonon DOS.	Figs. 10(*b*) and 11(*b*)
euphonic-show-sampling	Plots a 3D visualization of the distribution of **Q**-points over a sphere for the different spherical sampling schemes that are used in powder averaging. This tool shows the dimensionless sampling; in the *Euphonic* powder averaging routines these vectors are scaled by the desired |**Q**| to obtain reciprocal space coordinates.	Fig. 8
euphonic-optimise-dipole-parameter	The ‘dipole parameter’ determines the balance of real and reciprocal terms used in the Ewald sum for calculating the dipole correction (see Section 2.1.3). A higher value uses more reciprocal terms, and a lower value more real terms. Tuning this parameter can improve performance; this tool runs the interpolation for a few **Q**-points for a few different values of the parameter, and suggests an optimum value.	–

## Footnotes

1 The term ‘sloppy’ here means the chopper has relatively wide openings to maximize flux over a wide range of energies (particularly low energies <100 meV), in contrast to other choppers which are optimized for sharp resolution at specific (high) energies, but at the cost of lower transmission and flux.
