# Supplementary material for: Euphonic: inelastic neutron scattering simulations from force constants and visualization tools for phonon properties
Source: J Appl Crystallogr. 2022 Nov 29;55(Pt 6):1689–703. doi: 10.1107/S1600576722009256 (PMC9721333; doi:10.1107/S1600576722009256)
Supplement: Supplementary file 1 [file j-55-01689-sup2.pdf]

JOURNAL OF APPLIED CRYSTALLOGRAPHY

Volume 55 (2022)

Supporting information for article:

Euphonic: inelastic neutron scattering simulations from force constants and visualisation tools for phonon properties

Rebecca Fair, Adam Jackson, David Voneshen, Dominik Jochym, Duc Le, Keith Refson and Toby Perring

Supporting information for:
Euphonic: inelastic neutron scattering simulations from force constants and visualisation tools for phonon properties

Rebecca Fair[a], Adam Jackson[b], David Voneshen[a,c], Dominik Jochym[b], Duc Le[a], Keith Refson[a], and Toby Perring[a]

[a]ISIS Neutron and Muon Source, STFC Rutherford Appleton Laboratory, Harwell Campus, Didcot, Oxfordshire OX11 0QX, UK
[b]Scientific Computing Department, STFC Rutherford Appleton Laboratory, Harwell Campus, Didcot, Oxfordshire OX11 0QX, UK
[c]Department of Physics, Royal Holloway University of London, Egham, TW20 0EX, UK

1 Validation calculation

1.1 Intensity scaling

For the comparison of *Euphonic* [5] with *Ab2tds* [10] and *OClimax* [2], a scaling factor was calculated for each intensity map by averaging the ratio of the intensities in each bin with those in the corresponding bins in the *Euphonic* intensity map. Data points at energies less than 1 meV were first removed to eliminate acoustic mode data close to the gamma points (where the phonon intensity diverges) and afterwards any values that were smaller than 10^{-4} of the maximum intensity in the remaining portion of the map were removed to avoid potential numerical instabilities arising in the computation of very small intensities. The *Ab2tds* or *OClimax* intensity map was then normalised by the resulting scaling factor and the mean relative percentage difference (MRPD) was computed for the data that passed the same filtering criteria.

1.2 Symmetrisation of the Debye–Waller exponent

In all cases the grid used to calculate the Debye–Waller factor was a fully unfolded Monkhorst–Pack grid, i.e. the set of points was not symmetry reduced. It was found that using a symmetry reduced grid produces slightly different values for the Debye–Waller exponent W_κ than the full grid unless the Debye–Waller exponent is explicitly symmetrised so that it is invariant under symmetry operations. This would have made comparison between the different codes difficult as *Ab2tds* and *OClimax* appear to handle symmetry reduced grids differently, increasing the MRPDs depending on which grid type and symmetrisation settings are used. (*Euphonic* has the option to switch on or off Debye–Waller symmetrisation). An example of the MRPD increase for *Ab2tds* is shown in Table 1 for the case where a symmetry reduced grid was used as input and Debye–Waller symmetrisation was disabled in *Euphonic*. The MRPDs are higher than in the corresponding Table 3 in the main text, where a full grid was used. If the Debye–Waller symmetrisation is enabled again in *Euphonic* when using the reduced grid, the low MRPDs seen in Table 3 in the main text are recovered.

Table 1: Mean relative percentage difference between *Euphonic* and *Ab2tds* as in Table 3 in the main text, but using a symmetry reduced Monkhorst–Pack grid to calculate the Debye–Waller factor, and with Debye–Waller symmetrisation turned off in *Euphonic*

		Mean Relative Percentage Difference	
Material	**Q**-direction	*Euphonic* Interpolation	*CASTEP* Interpolation
Nb	$[h, h, 0]$	0.21	0.21
	$[2-k, k, 0]$	0.25	0.25
Quartz	$[h, -4, 0]$	1.89	1.89
	$[-3, 0, -l]$	0.27	0.23
$La_2Zr_2O_7$	$[-5, 7, -l]$	1.61	1.61
	$[h, -h, -2]$	1.15	1.15

2 Force constants calculation details

The input and output files for each of the following calculations are available at `https://doi.org/10.5281/zenodo.6620084` [4].

2.1 $La_2Zr_2O_7$

Calculations of the force constants were performed with DFT as implemented in *CASTEP* 17.21 [3]. Default ultrasoft pseudopotentials were used and the results presented used the Local Density Approximation (LDA) to exchange and correlation. A plane wave cut-off of 600 eV with electronic **k**-point sampling on a Monkhorst–Pack mesh of $4 \times 4 \times 4$ within the primitive cell was found to reduce the error in the forces to below 0.005

eV Å^{-1}. The structure and lattice parameters were relaxed with the quasi-Newton method [1] corrected for the finite basis set [6]. Force constants were calculated using the finite displacement/supercell method [7] within a single cubic unit cell.

2.2 Quartz

Original calculations were performed with *CASTEP* 6.1 using LDA exchange and correlation, optimised norm-conserving pseudopotentials [11], a plane-wave cutoff of 880 eV and a $5 \times 5 \times 4$ Monkhorst–Pack mesh for **k**-point sampling. The optimised lattice parameters were 4.852Å and 5.350Å. DFPT calculations also sampled using a $5 \times 5 \times 4$ grid with dipole-dipole model corrections applied. The resulting force constants were re-processed for this work using *CASTEP* 19.1.

2.3 Nb

Niobium calculations used the one-atom primitive cell with bcc lattice parameter of 3.25988Å, *CASTEP* 19.1, LDA exchange and correlation and a plane-wave cutoff of 900 eV. The NCP19 on-the-fly (OTF) library pseudopotential used is constructed with unfrozen 4s and 4p semi-core states. Electronic states were sampled using an $18 \times 18 \times 18$ Monkhorst–Pack grid and smeared using Gaussian broadening with a width of 0.5 eV. DFPT calculations were performed for a $9 \times 9 \times 9$ grid of **q**-points and the force constant matrix constructed by Fourier transforming on this grid.

2.4 Al

Force constants were obtained from a $4 \times 4 \times 4$ supercell of the cubic (conventional) unit cell using *Phonopy* [12] to implement and process a symmetrised finite-displacement method. The force calculator was *VASP* 5.4.4 [9], with the standard LDA PAW setup and parameters:

```
ENCUT = 600.000000
KSPACING = 0.100000
SIGMA = 0.200000
EDIFF = 1.00e-08
ALGO = fast
PREC = accurate
IBRION = -1
ISMEAR = 0
ISYM = 2
KPAR = 6
NCORE = 6
ADDGRID = .TRUE.
LASPH = .TRUE.
LREAL = .FALSE.
```

After **k**-point convergence and optimisation the unit cell has a lattice parameter of 3.984 207 Å.

2.5 Si

Force constants were obtained from a $5 \times 5 \times 5$ Γ-centered **Q**-point mesh sampling of a Si primitive cell, using DFPT with *CASTEP* 19.1, on-the-fly-generated norm-conserving pseudopotentials and LDA exchange-correlation functional with a "Precise" (266.9 eV) plane-wave basis set. The geometry was obtained by local optimisation in *CASTEP* from an initial structure generated with *Atomic Simulation Environment* (ASE) [8].

3 Powder-averaged calculation details and parameters

3.1 Al

The powder-averaging command was

```
euphonic-powder-map Al-444-lda.yaml --weighting coherent \
    --q-max 11 --asr --temperature 5 \
    --npts-density 400 --npts-min 500 --npts-max 20000 \
    --energy-broadening 1 --q-broadening 0.1 \
    --e-min -25 --e-max 60 --v-max 2 \
    --angle-range 3 135 --e-incident 60 \
    --no-widgets --style custom.mplstyle \
    --save-to al-simulated.png \
    --x-label '$|\mathbf{Q}|$ $(\AA^{-1})$' \
    --y-label 'Energy transfer (meV)'
```

3.2 Si

The powder-averaging command was

```
euphonic-powder-map Si-prim-555.json --weighting coherent \
    --q-max 12.5 --asr --temperature 300 \
    --npts-density 400 --npts-min 500 --npts-max 20000 \
    --energy-broadening 1 --q-broadening 0.1 \
    --e-min -40 --e-max 80 --v-max 5 \
    --angle-range 3 135 --e-incident 80 \
    --no-widgets --style custom.mplstyle \
    --save-to si-simulated.png \
    --x-label '$|\mathbf{Q}|$ $(\AA^{-1})$' \
    --y-label 'Energy transfer (meV)'
```

References

[1] Richard H. Byrd, Jorge Nocedal, and Robert B. Schnabel. "Representations of quasi-Newton matrices and their use in limited memory methods". en. In: *Mathematical Programming* 63.1 (Jan. 1994), pp. 129–156. ISSN: 1436-4646. DOI: 10.1007/BF01582063. URL: https://doi.org/10.1007/BF01582063 (visited on 01/11/2022).

[2] Y. Q. Cheng et al. "Simulation of Inelastic Neutron Scattering Spectra Using OCLIMAX". en. In: *Journal of Chemical Theory and Computation* 15.3 (Mar. 2019), pp. 1974–1982. ISSN: 1549-9618, 1549-9626. DOI: 10.1021/acs.jctc.8b01250. URL: http://pubs.acs.org/doi/10.1021/acs.jctc.8b01250 (visited on 03/25/2019).

[3] Stewart J. Clark et al. "First principles methods using CASTEP". en. In: *Zeitschrift für Kristallographie - Crystalline Materials* 220.5-6 (May 2005), pp. 567–570. ISSN: 2196-7105. DOI: 10.1524/zkri.220.5.567.65075. URL: https://www.degruyter.com/document/doi/10.1524/zkri.220.5.567.65075/html (visited on 01/11/2022).

[4] R. Fair et al. *Phonon calculations and measurements dataset for use with the Euphonic program.* July 2022. DOI: 10.5281/zenodo.6620084. URL: https://doi.org/10.5281/zenodo.6620084.

[5] Rebecca Fair et al. *Euphonic.* 2022. DOI: 10.5286/SOFTWARE/EUPHONIC. URL: https://doi.org/10.5286/SOFTWARE/EUPHONIC (visited on 06/13/2022).

[6] G P Francis and M C Payne. "Finite basis set corrections to total energy pseudopotential calculations". In: *Journal of Physics: Condensed Matter* 2.19 (May 1990), pp. 4395–4404. ISSN: 0953-8984, 1361-648X. DOI: 10.1088/0953-8984/2/19/007. URL: https://iopscience.iop.org/article/10.1088/0953-8984/2/19/007 (visited on 01/11/2022).

[7] W. Frank, C. Elsässer, and M. Fähnle. "Ab initio Force-Constant Method for Phonon Dispersions in Alkali Metals". In: *Physical Review Letters* 74.10 (Mar. 1995), pp. 1791–1794. DOI: 10.1103/PhysRevLett.74.1791. URL: https://link.aps.org/doi/10.1103/PhysRevLett.74.1791 (visited on 01/11/2022).

[8] Ask Hjorth Larsen et al. "The atomic simulation environment—a Python library for working with atoms". In: *Journal of Physics: Condensed Matter* 29.27 (July 2017), p. 273002. ISSN: 0953-8984, 1361-648X. DOI: 10.1088/1361-648X/aa680e. URL: https://iopscience.iop.org/article/10.1088/1361-648X/aa680e (visited on 05/20/2022).

[9] G. Kresse and J. Furthmüller. "Efficient iterative schemes for *ab initio* total-energy calculations using a plane-wave basis set". en. In: *Physical Review B* 54.16 (Oct. 1996), pp. 11169–11186. ISSN: 0163-1829, 1095-3795. DOI: 10.1103/PhysRevB.54.11169. URL: https://link.aps.org/doi/10.1103/PhysRevB.54.11169 (visited on 11/16/2021).

[10] Alessandro Mirone and Bjorn Wehinger. *Ab2tds*. 2013. URL: http://ftp.esrf.fr/scisoft/AB2TDS/Introduction.html.

[11] Andrew M. Rappe et al. "Optimized pseudopotentials". en. In: *Physical Review B* 41.2 (Jan. 1990), pp. 1227–1230. ISSN: 0163-1829, 1095-3795. DOI: 10.1103/PhysRevB.41.1227. URL: https://link.aps.org/doi/10.1103/PhysRevB.41.1227 (visited on 05/20/2022).

[12] Atsushi Togo and Isao Tanaka. "First principles phonon calculations in materials science". en. In: *Scripta Materialia* 108 (Nov. 2015), pp. 1–5. ISSN: 13596462. DOI: 10.1016/j.scriptamat.2015.07.021. URL: https://linkinghub.elsevier.com/retrieve/pii/S1359646215003127 (visited on 11/05/2020).
